# Antimicrobial activity and mechanistic insights of AMP-17 against drug-resistant *Pseudomonas aeruginosa* and its efficacy in wound infection management

**DOI:** 10.3389/fcimb.2025.1634825

**Published:** 2025-08-08

**Authors:** Ruyu Tao, Chunren Tian, Ting Su, Ruxia Cai, Dongxu Song, Na Zhao, Yuansan Lei, Zhenlong Jiao, Guo Guo

**Affiliations:** ^1^ Center of Laboratory Medicine, The Second Affiliated Hospital of Guizhou Medical University, Kaili, China; ^2^ School of Basic Medical Sciences, Guizhou Key Laboratory of Microbio and Infectious Disease Prevention & Control, Guizhou Medical University, Guiyang, China; ^3^ Key Laboratory of Environmental Pollution Monitoring and Disease Control, Ministry of Education, Guizhou Medical University, Guiyang, China; ^4^ Translational Medicine Research Center, Guizhou Medical University, Guiyang, China

**Keywords:** AMP-17, *Pseudomonas aeruginosa*, antimicrobial peptide, wound infection, mechanism of action

## Abstract

**Introduction:**

Chronic wound infections caused by drug-resistant bacteria have emerged as a global health challenge, affecting millions of patients annually and imposing a substantial economic and societal burden. However, current therapeutic approaches exhibit limited efficacy in treating drug-resistant wound infections, highlighting an urgent need for novel treatment strategies. Our previous studies have demonstrated that the *Musca domestica* antimicrobial peptide-17 (AMP-17) exhibits potent antibacterial activity, suggesting its potential as a promising anti-infective agent. Building on these findings, the present study isolated clinically relevant drug-resistant *Pseudomonas aeruginosa* (*P. aeruginosa*) strains and employed a combination of *in vitro* and *in vivo* experiments to systematically evaluate the efficacy of AMP-17 in combating drug-resistant infections and promoting wound healing. Furthermore, we preliminarily investigated the underlying mechanisms involved.

**Materials and methods:**

MIC/MBC of AMP-17 against drug-resistant *P. aeruginosa* were determined by microbroth dilution and agar spot assays. Biofilm inhibition/eradication was assessed via crystal violet staining, while swarming motility was tested on semi-solid agar. A murine wound infection model was established to evaluate the *in vivo* antimicrobial activity of AMP-17, and the levels of relevant cytokines were measured to preliminarily explore its anti-inflammatory mechanisms. Furthermore, the effects of AMP-17 on membrane permeability, proton motive force (PMF), pyocyanin production, and reactive oxygen species (ROS) levels in drug-resistant Pseudomonas aeruginosa were systematically investigated using scanning electron microscopy (SEM), confocal laser scanning microscopy (CLSM), and a multifunctional fluorescence microplate reader.

**Results and discussion:**

The results of this study demonstrate that AMP-17 exhibits significant antimicrobial activity against drug-resistant Pseudomonas aeruginosa. The underlying mechanisms primarily involve disruption of bacterial membrane integrity, alteration of proton motive force (PMF), increased intracellular ROS levels, and inhibition of bacterial motility, ultimately leading to bacterial cell death. Additionally, AMP-17 shows promising efficacy in inhibiting biofilm formation and eradicating mature biofilms of drug-resistant *P. aeruginosa*. In a murine wound infection model, AMP-17 displayed potent *in vivo* antimicrobial activity, significantly reducing bacterial load and downregulating pro-inflammatory cytokine expression, thereby effectively promoting wound healing. Collectively, these findings highlight the potential of AMP-17 as a promising therapeutic agent for combating drug-resistant *P. aeruginosa* infections and enhancing wound healing.

## 1 Introduction

*P. aeruginosa* belongs to non-fermentative Gram-negative bacilli and is one of the most common conditional pathogens in hospital-acquired infections (Tenover et al., 2022), which can cause pneumonia, urinary tract infections, and wound infections (de Sousa et al., 2021; Uberoi et al., 2024). The overuse and misuse of antibiotics is associated with an explosion of antimicrobial drug resistance. *P. aeruginosa* was also included in the list of global priority pathogens posing the greatest threat to human health by the World Health Organization (WHO) in 2017 for multi-drug resistance and pan-drug resistance (Tacconelli et al., 2018). The CDC report on antibiotic resistance warns us that we are already living in a post-antibiotic era and that the development of new antibiotics is being challenged (Wang et al., 2020; Flynn and Guarner, 2023). Currently, more than 700,000 people die each year from “superbug” infections. If no action is taken, the number of deaths could increase to 10 million by 2050 (de Oliveira et al., 2024). A U.S. retrospective analysis revealed that bloodstream infections with difficult-to-treat strains increased adjusted mortality by 40%, while a prospective Italian cohort demonstrated significantly higher 30-day mortality in carbapenem-resistant *P. aeruginosa* infections versus susceptible strains (Miller and Arias, 2024). These escalating regional epidemics highlight the urgent need for novel antimicrobial strategies. Therefore, research and development of novel antimicrobial drugs to address the growing problem of *P. aeruginosa* resistance is imminent.

*Musca domestica* demonstrates remarkable adaptability to highly contaminated environments with elevated microbial loads (Manniello et al., 2021). Even under such extreme conditions, this species maintains an efficient innate immune defense system, in which antimicrobial peptides (AMPs) serve as crucial components of its immunological mechanisms (Zhou et al., 2024). AMPs are some small molecular peptides with antimicrobial activity in living organisms, which are produced when the organism is induced by infection or immune stimulation and have a unique antimicrobial mechanism and antibacterial activity against bacteria, fungi and viruses. With the misuse of antibiotics and the emergence of super-resistant bacteria, insect antimicrobial peptides have become an important resource for the design and development of novel antimicrobial drugs.

Compared to traditional antibiotics, AMPs exhibit a broader spectrum of antimicrobial activity and employ a unique membrane-disrupting mechanism that reduces the risk of bacterial resistance development (Ji et al., 2024). Furthermore, AMPs demonstrate distinct advantages in wound infection management, as certain anti-inflammatory peptides can downregulate pro-inflammatory cytokines, thereby mitigating chronic inflammation and promoting healing (Li et al., 2025). With their broad-spectrum efficacy, low propensity for resistance induction, and wound-healing properties, AMPs represent a promising therapeutic candidate for combating drug-resistant wound infections, holding considerable potential for clinical applications.

In previous investigations, our research group conducted whole transcriptome sequencing of third-instar larvae of *Musca domestica*, identifying a gene significantly involved in the immune defense mechanisms of this species. Utilizing genetic engineering techniques, we successfully synthesized a recombinant protein designated as AMP-17. Subsequent studies have elucidated the spatiotemporal expression profile and antifungal properties of AMP-17 (Guo et al., 2017; Sun et al., 2022). Although we refer to this molecule as AMP-17 (Antimicrobial Peptide-17) in accordance with its historical designation, it should be noted that based on its molecular weight, it could alternatively be classified as an antimicrobial protein. Intriguingly, our preliminary experiments demonstrated that AMP-17 exhibits potent antibacterial activity against both standard strains and clinically isolated multidrug-resistant *P. aeruginosa*. Nevertheless, the precise molecular pathways through which AMP-17 exerts its antimicrobial effects remain to be fully elucidated, and its underlying antibacterial mechanisms warrant further investigation. Therefore, this study aimed to evaluate the *in vitro* antimicrobial activity of AMP-17 against *P. aeruginosa* CMCC 10104 and its multidrug-resistant clinical isolates, and further validate the *in vivo* antibacterial efficacy of AMP-17 by establishing a wound infection model. By investigating the morphological alterations, changes in membrane permeability, and intracellular ROS levels following AMP-17 treatment, we preliminarily elucidated the antibacterial mechanism of AMP-17 against *P. aeruginosa*. The findings of this study will provide a theoretical foundation for developing novel antimicrobial peptide antibiotics and offer new strategies for the clinical treatment of multidrug-resistant *P. aeruginosa* infections.

## 2 Materials and methods

### 2.1 Experimental strains and culture

The clinical drug-resistant strains (*P. aeruginosa* 01~10) were obtained from a tertiary care hospital located in Kaili City, Guizhou Province, with all isolates originating from hospitalized patients. The *P. aeruginosa* CMCC 10104 was obtained from the Key and Characteristic Laboratory of Modern Pathogen Biology at Guizhou Medical University. All strains were preserved in 15% glycerol at -80°C and cultured in LB medium at 37°C with shaking at 220 rpm until reaching the logarithmic growth phase.

### 2.2 Antimicrobial peptide

The preparation of AMP-17 was carried out according to the method described by (Yang et al., 2022). The protein was purified using a Ni-NTA column, and the concentration of AMP-17 was determined using a BCA protein assay kit.

### 2.3 Antibacterial assays

Referring to the judgment criteria in the American Committee for Clinical Laboratory Standardization (CLSI), the subculturing of bacterial broth in MHB medium was repeated twice. One monoclonal colony was selected, and inoculated into 5 mL of LB liquid medium until the logarithmic growth phase. The bacterial suspension was adjusted to a McFarland turbidity standard using a densitometer, followed by conversion to colony-forming units (CFU). The bacterial concentration was then standardized to 1×10^6^ CFU/mL using 1× PBS for subsequent experiments. The concentration of AMP-17 and 1.0×10^6^ CFU/mL bacterial suspension were incubated in 96-well plates at 37°C for 16 h. The MIC value was determined as the concentration of the drug in the wells with no visible bacterial growth. The concentration of the antimicrobial peptide corresponding to no bacterial growth on the LB plate was used as the MBC. 100 μL of bacterial suspension was diluted to the appropriate concentration from the wells judged as MIC and the wells higher than the concentration of the drug in the wells, and then evenly coated on LB plates and incubated at 37°C for 24 h. MHB medium was used for the blank control, MHB medium with bacterial suspension for the negative control, and 4 μg/mL of polymyxin B for the positive control.

### 2.4 Detection of growth curve and time-kill kinetics

The growth curve analysis of bacteria was based on previous methods with modifications (Boulaamane et al., 2024). The bacterial suspension (1.0×10^6^ CFU/mL) was added into 96-well plates and incubated with different concentrations (1/2~2 ×MIC) of AMP-17 at 37°C for 24 h. The absorbance values at 600 nm were measured hourly, and the results were recorded and the growth curves were plotted. The peptide antibiotic Polymyxin B (2×MIC) was used as the positive control, MHB as the blank control, and MHB medium with bacterial suspension was used as the negative control.

The time-kill kinetics experiment was performed as described in previous study (Lasko et al., 2022). AMP-17 (1/2~2 ×MIC) was incubated with 1×10^6^ CFU/mL of *P. aeruginosa* CMCC 10104 and 04 suspension at 37°C for 24 h. When incubated for 0, 1, 2, 4, 6, 8, 10, 12, 24 h, 10 μL of the AMP-17-activated suspension was serially diluted in MHB medium at ten-fold dilution to the appropriate concentration. 5 μL of the dilution was pipetted onto LB plates, and the colonies were counted and the time-kill curve was plotted after the colonies had grown.

### 2.5 Effects of salt and serum on AMP-17 activity

To investigate the activity of AMP-17 under salt conditions and in the presence of serum, we performed MIC assays using the microbroth dilution method. *P. aeruginosa* CMCC10104 and 04 were inoculated in Mueller-Hinton broth (MHB) containing gradient concentrations of AMP-17. The culture media were supplemented with either 150 mM NaCl, 4.5 mM KCl, 2.5 mM CaCl_2_, 10% murine serum, or fetal bovine serum (FBS). The inoculated 96-well plates were incubated at 37°C for 16 h, followed by MIC determination to evaluate potential alterations in the antibacterial activity of AMP-17.

### 2.6 Bacterial motility assay

The motility assay refers to the theory of (Fang et al., 2022). *P. aeruginosa* CMCC 10104 and 04 in logarithmic growth phase was prepared into 1.0 × 10^6^ CFU/mL bacterial suspension and set aside. When the medium temperature was lowered to 50°C, AMP-17 at final concentrations of 1/4 ×MIC and 1/2 ×MIC was added to the medium, and 2 μL of the suspension was pipetted onto 0.3% (w/v) agar medium containing tryptic peptone (10 g/L), NaCl (10 g/L) and yeast extract (5 g/L). The plates were allowed to dry naturally and then incubated orthotropically at 37°C for 24 h. The bacterial motility area was measured and the effect of AMP-17 on the motility of *P. aeruginosa* was assessed.

### 2.7 Biofilm formation inhibition experiment

Biofilm formation is a prevalent phenomenon in wound environments and constitutes a significant barrier to the efficacy of antimicrobial agents. In order to evaluate the potential of AMP-17 against biofilms formation *in vitro*, an antibiofilm assay was performed as follows (Rajan et al., 2023). A bacterial suspension of *P. aeruginosa* CMCC10104 and 04 (1.0×10^6^ CFU/mL) was prepared in Tryptic Soy Broth (TSB) and co-cultured with serial dilutions of AMP-17 (1/2~2 ×MIC) in 96-well microtiter plates at 37°C for 24 hours. Sterile TSB served as a blank control, while MHB with bacteria acted as a negative control. After incubation, non-adherent cells were removed by aspirating the supernatant and washing twice with sterile phosphate-buffered saline (PBS). The remaining biofilms were methanol-fixed (15 min), air-dried, and stained with 0.1% (w/v) crystal violet for 30 min at room temperature. Excess stain was washed off with PBS, and biofilm-associated dye was solubilized in anhydrous ethanol (200 µL/well) at 37°C for 30 min. The absorbance at 595 nm was measured using a microplate reader to quantify biofilm biomass.

### 2.8 Mature biofilm removal experiment *in vitro*

A bacterial suspension of *P. aeruginosa* CMCC 10104 and 04 at a concentration of 1.0×10^6^ CFU/mL was prepared and incubated at 37°C for 24 h. Following the removal of nonadherent bacteria by washing with sterile phosphate-buffered saline, 200 μL of AMP-17 at varying concentrations were added to the wells and incubated at 37°C for 2 hours. Subsequently, the biofilm biomass was quantified by measuring the absorbance at 595 nm after staining with crystalline violet. TSB served as the blank control, whereas MHB containing the bacterial suspension functioned as the negative control.

To visualize biofilm eradication more comprehensively, the experiment refers to the theory of (Tchatchiashvili et al., 2024). The biofilms were dual-stained with 10 μM propidium iodide (PI) and 5 μM SYTO 9. PI selectively labels dead or membrane-compromised bacterial cells, while SYTO 9 stains both live and dead cells, enabling the differentiation of cell viability within the biofilm. The stained biofilms were then subjected to imaging using a CLSM with excitation wavelengths of 488 nm and 561 nm for SYTO 9 and PI, respectively.

### 2.9 Determination of pyocyanin

The quantification of pyocyanin was performed by the method of (Gasu et al., 2019). A bacterial suspension of *P. aeruginosa* CMCC 10104 was prepared at a concentration of 1.0×10^6^ CFU/mL. AMP-17 was then added to achieve final concentrations of 1/2 to 2 ×MIC, and the mixture was incubated at 37°C for 24 hours. Following incubation, the culture was centrifuged at 5000 rpm for 10 minutes to obtain the supernatant. Prior to pyocyanin measurement, bacterial concentrations in all samples were adjusted to 1 × 10^6^ CFU/mL through turbidimetric assessment and plate counting to ensure standardized comparisons. 5 mL of chloroform was added to the supernatant, and after thorough vortexing, the mixture was allowed to stand for 8 minutes. The sample was then centrifuged at 4500 rpm for 10 minutes, and the lower, blue-colored chloroform phase was collected. Next, 1 mL of 0.2 mol/L hydrochloric acid was added to the collected phase, and the mixture was allowed to stand for 30 minutes with intermittent vortexing until extraction was complete. After a final centrifugation at 4500 rpm for 8 minutes, the resulting pink supernatant was collected. The pyocyanin concentration (μg/mL) was calculated using the following formula, where 17.072 represents the molar extinction coefficient of pyocyanin at 520 nm:


$$\text{pyocyanin content}\;(\text{mg}/\text{mL})={\text{ OD}}_{520}\;\times \;17.072$$

### 2.10 Morphological characterization by electron microscopy

Bacterial suspension of *P. aeruginosa* CMCC 10104 and 04 were prepared at 1.0×10^6^ CFU/mL and treated with AMP-17 at a final concentration of 2 ×MIC. The sample was incubated at 37°C for 8 hours, then centrifuged at 3000 g for 10 minutes to pellet the cells. The pellet was washed once with sterile PBS, discarding the supernatant thereafter. The cells were resuspended in 2.5% glutaraldehyde and fixed overnight at 4°C in the dark. Following fixation, the cells underwent sequential dehydration in graded ethanol solutions (30%, 50%, 70%, 80%, 90%, 95%, and 100%). The dehydrated samples were dried under reduced pressure and subsequently sputter-coated with gold using an ion sputterer. SEM was then employed to observe the cellular morphology, and representative micrographs were collected. A PBS-treated group served as the negative control.

For TEM observation, the samples were prepared in a manner analogous to that used for scanning electron microscopy. Briefly, the samples were centrifuged at 3000 g for 10 minutes, and the supernatant was discarded. The pellet was then fixed in 2.5% glutaraldehyde at 4°C overnight in the dark. Following this primary fixation, the samples were post-fixed in 1% ephorbic acid at 4°C for 2 hours. Subsequently, the specimens underwent graded dehydration in ethanol for 20 minutes per concentration step, followed by two washes in 100% acetone, each lasting 15 minutes. The dehydrated samples were then embedded in Epon 812 by immersion. Ultrathin sections were prepared, double-stained with uranyl acetate and lead nitrate, and examined using TEM, with micrographs collected.

### 2.11 Membrane fluidity

To assess membrane fluidity (Verstraeten et al., 2021), the bacterial suspension of *P. aeruginosa* CMCC 10104 and 04 were adjusted to a concentration of 1.0×10^6^ CFU/mL. Laurdan dye was introduced to achieve a final concentration of 10 μM, and the mixture was incubated at 37°C for 1 hour. Following incubation, the cells were washed once with sterile PBS. Subsequently, 100 μL aliquots of the bacterial suspension were exposed to AMP-17 at concentrations of 1/2 to 2 ×MIC for 1 hour. Fluorescence intensity was measured using a multifunctional microplate reader, with an excitation wavelength of 350 nm and emission wavelengths of 435 nm and 490 nm. The Laurdan Generalized Polarization was calculated using the following equation:


$$\text{Laurdan GP }=\;\left({\text{I}}_{435}-{\text{ I}}_{490}\right)\;/\;\left({\text{I}}_{435}\;+{\text{ I}}_{490}\right)$$

### 2.12 AMP-17 effects on Transmembrane potential (Δψ) and ΔpH in *P. aeruginosa*

The transmembrane potential refers to the theory of (Song et al., 2021). Various concentrations of AMP-17 were introduced to the 1.0×10^6^ CFU/mL bacterial suspension, followed by the addition of DiSC_3_ (5) dye to a final concentration of 0.5 μM. The mixture was incubated in the dark at 37°C for 15 minutes. Subsequently, 100 μL of the treated suspension was transferred to a black 96-well microplate. Fluorescence intensity was measured using a multifunctional fluorometer, with excitation and emission wavelengths set at 622 nm and 670 nm, respectively. Fluorescence changes were continuously monitored and recorded over a 1-hour period.

To detect changes in ΔpH in *P. aeruginosa*, the 1.0×10^6^ CFU/mL bacterial cells were then incubated with 1 μM of the pH-sensitive fluorescent probe BCECF-AM at 37°C for 30 minutes under low-light conditions (Kim, 2024). Following this incubation, the cells were treated with varying concentrations of AMP-17 at 37°C for 1 hour, again under dark conditions. Fluorescence intensity was measured using a multifunctional fluorometric plate reader, with excitation and emission wavelengths set at 488 nm and 535 nm, respectively.

### 2.13 Proportion of live and dead bacteria

*P. aeruginosa* CMCC 10104 was cultured to a concentration of 1.0×10^6^ CFU/mL and incubated with AMP-17 at final concentrations of 1/2 to 2 ×MIC at 37°C for 8 hours. A control group was treated with an equal volume of sterile 1×PBS under the same conditions. Following incubation, 5 μM SYTO 9 and 10 μM PI were added to each sample, and the mixtures were incubated at 37°C for 15 minutes in the dark. Subsequently, bacterial suspension was placed on microscope slides. Fluorescence images were captured using a CLSM.

### 2.14 Membrane permeability

To assess outer membrane permeability, we referred to the method of (Yang et al., 2023). The 1.0×10^6^ CFU/mL bacterial suspension was incubated with AMP-17 (at final concentrations of 1/2~2 ×MIC) at 37°C in a 96-well black microplate. Following this, N-phenyl-1-naphthylamine (NPN) dye was added to each well to a final concentration of 10 μM, and the mixtures were incubated in the dark. Fluorescence intensity was measured using a multifunctional microplate reader, with excitation and emission wavelengths set at 350 nm and 420 nm, respectively.

The inner membrane permeability experiment was performed as described in previous study (Mikkola et al., 2019). The 1.0×10^6^ CFU/mL bacterial suspension was incubated with PI at a final concentration of 10 μM for 15 minutes in the dark. Subsequently, AMP-17 was added to achieve final concentrations of 1/2 to 2 ×MIC, and the mixture was incubated at 37°C for 1 hour in 96-well black microplates. Fluorescence intensity was measured using a multifunctional microplate reader, with excitation and emission wavelengths set at 622 nm and 670 nm, respectively.

### 2.15 Reactive oxygen level detection

To evaluate ROS production, the experiment was performed as described in previous study (Baker et al., 2021). The 1.0×10^6^ CFU/mL bacterial suspension was incubated with AMP-17 (the final concentrations of 1/2~2 ×MIC). Subsequently, 2’,7’-dichlorodihydrofluorescein diacetate (DCFH-DA) was added to a final concentration of 10 μM, and fluorescence intensity was continuously monitored at 37°C for 1 hour. Real-time fluorescence measurements were conducted using a multifunctional microplate reader, with excitation and emission wavelengths set at 485 nm and 528 nm, respectively.

### 2.16 Establishment of a mice wound model

To establish a mouse wound model, female Balb/c mice (6 weeks old, 18 ~ 20 g) were purchased from SPF Biotechnology Co., Ltd (Beijing, China). The animals were housed under a standard 12-hour light/12-hour dark cycle. All animal experiments were conducted in accordance with the Ethical Principles in Animal Research adopted by Guizhou Medical University and were approved by the Institutional Animal Care and Use Committee (The animal ethics review form number: No. 2302013).

After anesthetizing the mice, full-thickness oval wounds with a long axis of 10 mm were created on their backs using a puncher. Subsequently, 100 μL of *P. aeruginosa* solution (1.0 × 10^8^ CFU/mL) was applied to induce infection. The wound infection model was established by maintaining continuous infection for 2 days. The mice were then randomly divided into three groups (n = 6): control, vehicle and AMP-17. Wounds not infected with *P. aeruginosa* and treated with 100 μL volume of normal saline were designated as the control group, while wounds infected with *P. aeruginosa* and treated with the same volume of normal saline were designated as the vehicle group. AMP-17 was administered at a concentration of 2 ×MIC. A 100 μL volume of the respective sample was applied dropwise to the wound surface using a 1 mL syringe. Infected wounds received daily treatments for 7 days (100 μL once a day), with daily monitoring of wound changes. After 11 days, the mice were sacrificed, and the healing effects of each group were evaluated through hematoxylin-eosin (H&E) staining and Masson staining.

Wound tissue was collected in a sterile environment, weighed, and homogenized in 1 mL of normal saline. Serial dilutions were prepared, and the homogenate was inoculated onto LB solid culture plates, which were incubated for 24 hours at 37 °C. The bacterial load on the wound surface was calculated using the formula:


Bacterial load (CFU/g) = single colony count × dilution ratio/tissue mass (g)

Additionally, the wound tissue was homogenized in RIPA buffer for 5 minutes at 4°C. The levels of vascular endothelial growth factor (VEGF), interleukin-6 (IL-6), and tumor necrosis factor-alpha (TNF-α) in the supernatant were determined using ELISA kits.

### 2.17 Statistical analysis

The experiment was performed with three biological replicates per group. All data were processed by Graph Pad Prism 8.0.1 and expressed as mean ± standard deviation. T-test was used to analyze the differences between the two groups, and *P*< 0.05 was considered significant. The wound area was measured and calculated using ImageJ software.

## 3 Results

### 3.1 Detection of antibacterial activity of AMP-17 against *P. aeruginosa*

Ten multidrug-resistant *P. aeruginosa* strains were collected from clinical samples and exhibited varying degrees of resistance to five commonly used antibiotics: cephalosporins, carbapenems, aminoglycosides, quinolones, and peptides (**Table 1**). AMP-17 showed 1 ×MIC values of 25 µg/mL and MBC of 50 µg/mL against both the standard strain *P. aeruginosa* CMCC 10104 and the clinical isolates. Subsequent experiments were conducted using *P. aeruginosa* 04 as the representative clinical strain, selected based on its extensive multidrug resistance profile among the tested isolates.

**Table 1:** MIC values of several antimicrobial agents against *P. aeruginosa*.

*P. aeruginosa* Strains	Sample source types	MIC (μg/mL)							MBC (μg/mL)
		AMP-17	CAZ	MEM	PB	SCF	COL	LVX	AMP-17
CMCC 10104	lab	25	2 (S)	≤0.25 (S)	≤2 (S)	≤8 (S)	2 (I)	0.5 (S)	50
01	sputum	25	≥64 (R)	8 (R)	≤2 (S)	≥64 (R)	≤0.5 (S)	2 (I)	50
02	sputum	25	≥64 (R)	≥16 (R)	≤2 (S)	≥64 (R)	≤0.5 (S)	0.25 (S)	50
03	urine	25	≥64 (R)	8 (R)	≤2 (S)	≥64 (R)	≤0.5 (S)	1 (S)	50
04	wound exudate	25	≥64 (R)	≥16 (R)	≤2 (S)	≥64 (R)	4 (R)	≥8 (R)	50
05	wound exudate	25	16 (I)	≥16 (R)	≤2 (S)	≥64 (R)	≤0.5 (S)	2 (I)	50
06	wound exudate	25	16 (I)	≥16 (R)	≤2 (S)	≥64 (R)	≤0.5 (S)	4 (R)	50
07	wound exudate	25	2 (S)	4 (I)	≤2 (S)	≤8 (S)	≤0.5 (S)	2 (I)	50
08	sputum	25	32 (R)	8 (R)	≤2 (S)	≤8 (S)	≤0.5 (S)	1 (S)	50
09	sputum	25	32 (R)	8 (R)	≤2 (S)	32 (I)	≤0.5 (S)	≥8 (R)	50
10	sputum	25	≥64 (R)	≥16 (R)	≤2 (S)	≥64 (R)	≤0.5 (S)	1 (S)	50

CAZ, ceftazidime; MEM, meropenem; PB, polymyxin B; SCF, cefoperazone/sulbactam; COL, colistin; LVX, levofloxacin; I, intermediate; S, susceptible; R, resistant.

### 3.2 Bactericidal activity of AMP-17

**Figure 1:**
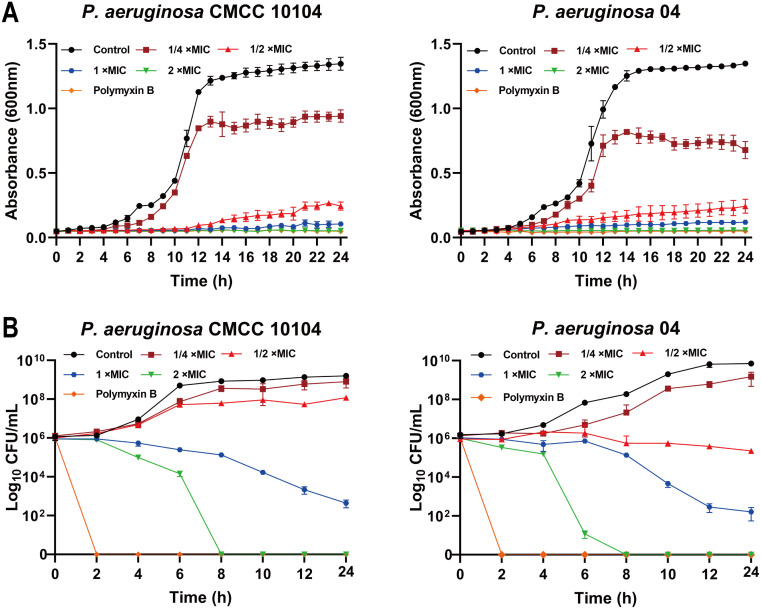
AMP-17 is an antimicrobial peptide exhibiting potent bactericidal activity. **(A)** The growth curve of *P. aeruginosa* CMCC 10104 and 04 were recorded over a 24 h incubation by measuring the optical density at 600 nm in the presence or absence of AMP-17 at concentrations ranging from 1/2 to 2 ×MIC. **(B)** The time-kill kinetics of AMP-17 against *P. aeruginosa* CMCC 10104 and 04 were determined by incubating bacterial cells with AMP-17 (1/2 to 2 ×MIC) for 24 hours, with colony counts performed every 2 hours.

The growth curve of *P. aeruginosa* CMCC 10104 and 04 without AMP-17 treatment was “S” shaped, and the bacterial growth reached the stationary phase after 16 h. Treatment with 1 ×MIC of AMP-17 inhibited bacterial growth within 10 hours, with only marginal OD_600_ increase observed by 24 hours. In contrast, 2×MIC treatment completely suppressed growth throughout the 24-hour incubation period. (**Figure 1A**). Time-kill kinetics experiments confirmed that AMP-17 exhibits bactericidal effects in a concentration- and time-dependent manner. At a concentration corresponding to 2 ×MIC, complete eradication of the bacterial population was achieved within 8 hours, and this inhibitory effect persisted for an additional 16 hours without any evidence of regrowth (**Figure 1B**). Notably, at the 6-hour time point, AMP-17 (2 ×MIC) demonstrated significantly enhanced bactericidal activity against the drug-resistant *P. aeruginosa* strain 04 compared to the reference strain CMCC10104, with the bacterial load being reduced by 3.091 log_10_ CFU/mL.

Notably, systematic evaluation of the salt and serum stability of AMP-17 revealed the following findings (**Table 2**). The MIC of AMP-17 demonstrated remarkable stability, showing no significant alterations in the presence of physiological concentrations (150 mM NaCl, 4.5 mM KCl, or 2.5 mM CaCl_2_) compared to the control group. Importantly, under simulated physiological conditions using culture media supplemented with 10% FBS or murine serum, AMP-17 maintained antimicrobial efficacy, with no change in MIC values.

**Table 2:** Effects of salt and serum on AMP-17 against *P. aeruginosa*.

Treatments	AMP-17 (MIC, μg/mL)	
	*P. aeruginosa* CMCC 10104	*P. aeruginosa* 04
150 mM NaCl	25	25
4.5 mM KCl	25	25
2 mM CaCl_2_	25	25
10% Fetal bovine serum	25	25
10% Murine serum	25	25

### 3.3 Bacterial motility assay

**Figure 2:**
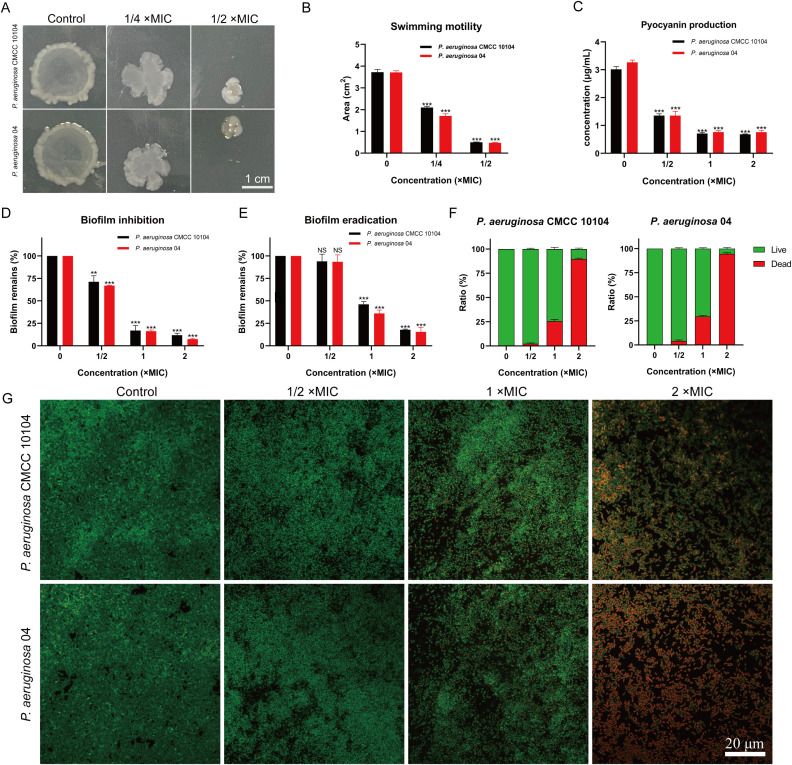
Effect of AMP-17 on *P. aeruginosa* CMCC 10104 and 04 bacterial motility and biofilm. **(A)** Image showing the colony area. **(B)** Measurement of colony area from the image. **(C)** Effect of AMP-17 on pyocyanin production. **(D)** Inhibition of biofilm formation by various concentrations of AMP-17. **(E)** Disruption of mature biofilms by different concentrations of AMP-17. **(F)** Live/Dead bacterial ratio in biofilm. **(G)** Confocal laser scanning microscopy images illustrating the effect of AMP-17 on mature *P. aeruginosa* CMCC 10104 and 04 biofilms. Statistical significance was determined by comparing each treatment group to the control group, with *P* values indicated as follows: **P < 0.01 and ***P < 0.001.

To investigate the effect of AMP-17 on *P. aeruginosa* motility, we utilized semisolid agar plates (**Figure 2A**). Bacterial migration was significantly reduced in the presence of AMP-17 after 24 hours of incubation. Notably, compared to the untreated control, exposure to AMP-17 at 1/4 ×MIC concentration induced reductions in swarming motility areas of *P. aeruginosa* CMCC10104 and strain 04 by 47.63% and 48.91%, respectively. Upon escalating the AMP-17 concentration to 1/2 ×MIC, both strains exhibited remarkably diminished motility zones with more pronounced inhibition rates of 88.04% and 90.26% (**Figure 2B**). These findings collectively suggest that AMP-17 may exert its antimicrobial effects through suppression of bacterial motility mechanisms. These findings suggest that AMP-17 impairs bacterial motility, thereby enhancing its bactericidal activity.

### 3.4 Pyocyanin content assay

Pyocyanin, a virulence factor produced by *P. aeruginosa*, plays a significant role in disrupting the host defense system and contributes to the pathogenesis of infections. As shown in **Figure 2C**, increasing concentrations of AMP-17 led to a significant reduction in pyocyanin production across all groups compared to the untreated strains. These findings suggest that AMP-17 effectively inhibits pyocyanin production, thereby potentially reducing the virulence of *P. aeruginosa* and aiding in infection control.

### 3.5 Effect of AMP-17 on the biofilm

Biofilms serve as protective barriers for bacteria, shielding them from antibiotic-induced eradication and contributing to the development of bacterial resistance. As illustrated in **Figure 2D**, the formation of *P. aeruginosa* biofilm was effectively inhibited by 1/2 ×MIC of AMP-17. Significantly, no statistically discernible eradication of mature biofilms was observed compared to the untreated control at 1/2×MIC (**Figure 2E**). However, AMP-17 at concentrations (1 and 2 ×MIC) elicited significant structural disintegration of biofilms.

To assess the bactericidal activity against biofilm-resident viable cells, we conducted SYTO9/PI dual fluorescence staining with confocal laser scanning microscopy and evaluated the proportion of live to dead bacteria within the biofilm (**Figures 2F, G**). Results revealed a dose-dependent intensification of red fluorescence (indicative of membrane-compromised cells) concomitant with diminished green fluorescence (viable cells), demonstrating that AMP-17 not only disrupts biofilm architecture but also induces substantial bacterial mortality within the biofilm.

### 3.6 Morphological changes induced by AMP-17

**Figure 3:**
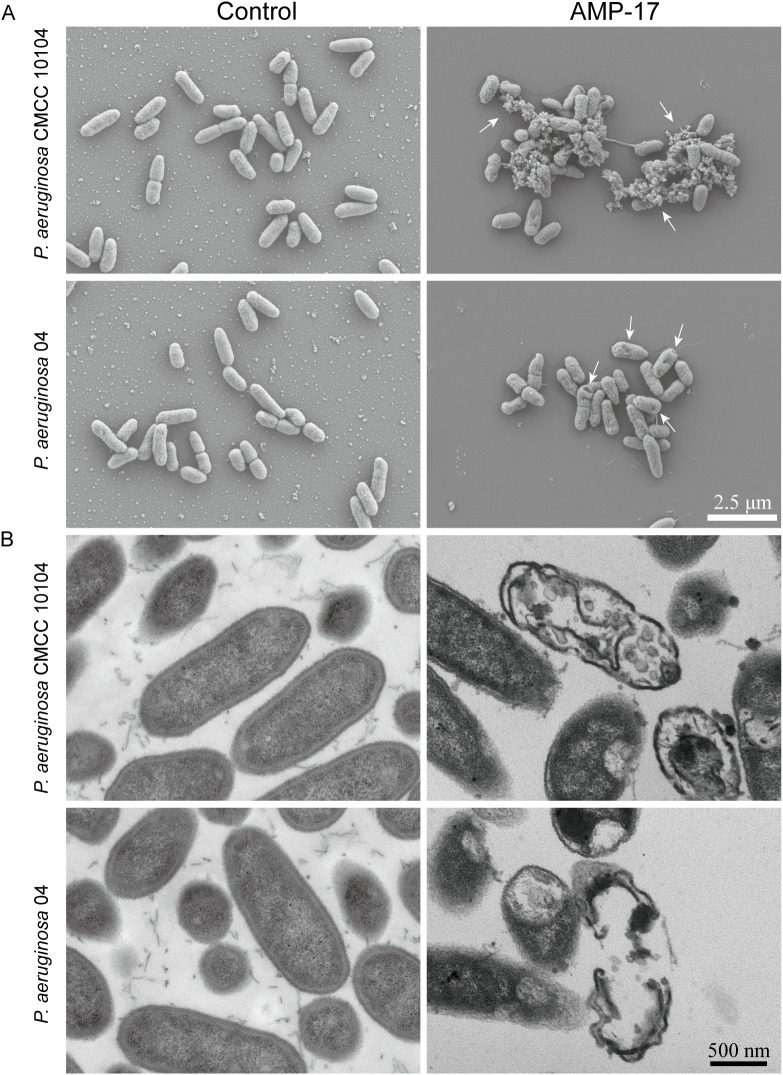
SEM and TEM images of *P. aeruginosa* CMCC 10104 and 04. **(A)** SEM images. **(B)** TEM images. *P. aeruginosa* treatment with AMP-17 (2 ×MIC) for 8 **(h)** White arrows indicate cell lysis products.

SEM revealed that untreated *P. aeruginosa* cells exhibited intact morphology with smooth, undamaged surfaces (**Figure 3A**). Following 24-hour treatment with 2×MIC AMP-17, both *P. aeruginosa* CMCC 10104 and 04 strains exhibited distinct ultrastructural alterations. SEM analysis revealed localized membrane rupture accompanied by cytoplasmic efflux in CMCC 10104 cells, whereas 04 cells displayed characteristic depression-like pores distributed across the bacterial surface. These morphological modifications suggest that AMP-17 may compromise cellular integrity through disruption of membrane architecture in *P. aeruginosa*.

TEM analysis further predicted the membrane-targeting effects of AMP-17. Untreated controls maintained characteristic membrane architecture with preserved cytoplasmic density and organelle organization (**Figure 3B**). In contrast, AMP-17-treated cells (2 ×MIC) exhibited discontinuous membrane continuity, accompanied by periplasmic space dilation. Notably, cytoplasmic content redistribution and nucleoid material dispersion were observed in treated specimens. These ultrastructural perturbations collectively indicate simultaneous disruption of membrane architecture and intracellular organization.

### 3.7 Effect of AMP-17 on membrane permeability

**Figure 4:**
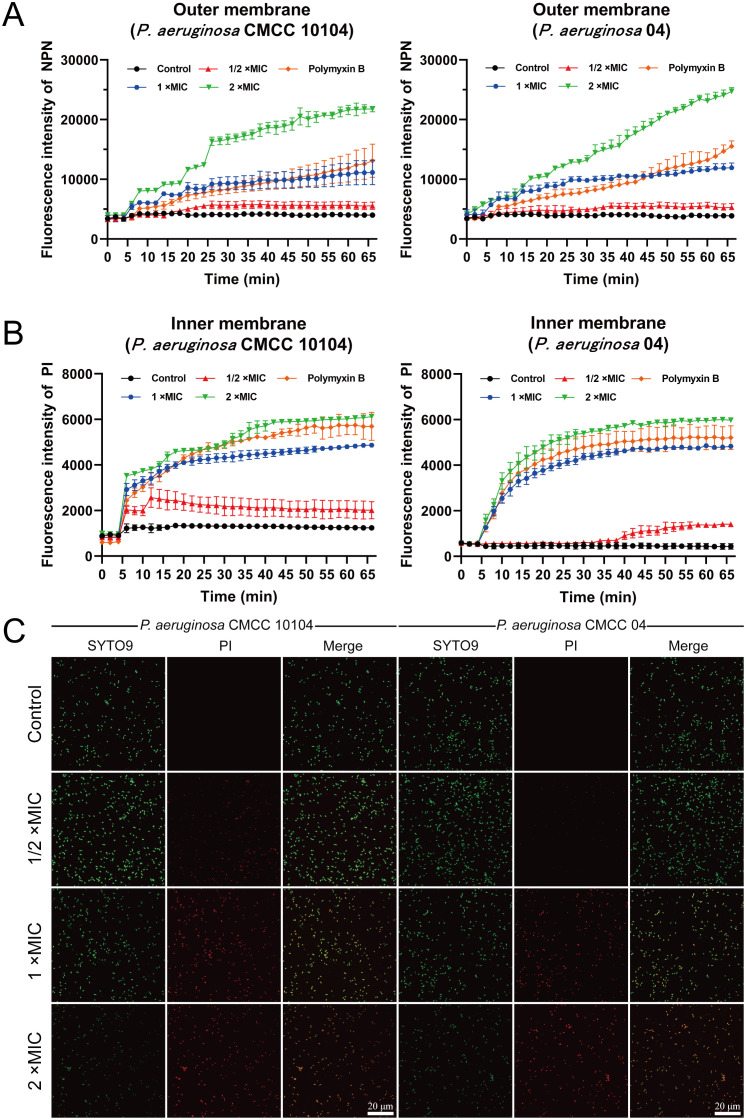
Effect of AMP-17 on membrane permeability of *P. aeruginosa* CMCC 10104 and 04. **(A)** Effect of AMP-17 on outer membrane permeability. **(B)** Effect of AMP-17 on inner membrane permeability. **(C)** CLSM images show the proportion of live and dead bacteria.

Fluorescence analysis using the NPN probe revealed modulatory effects of AMP-17 on the outer membrane permeability of *P. aeruginosa.* The control group exhibited minimal fluorescence signal fluctuations during the initial 5-minute monitoring period (**Figure 4A**), whereas AMP-17-treated samples demonstrated a progressive increase in fluorescence intensity, suggesting outer membrane permeability alterations. Parallel experiments with PI staining showed time-dependent enhancement of fluorescence signals (**Figure 4B**), correlating with modifications in inner membrane permeability. Live/dead cell viability assays (**Figure 4C**) revealed concentration-dependent responses to AMP-17 treatment. Bacterial samples treated with 1/2×MIC AMP-17 showed limited red fluorescence emission, whereas 1×MIC exposure elevated red fluorescence intensity relative to untreated controls, suggesting compromised membrane integrity. At 2×MIC concentration, the entire bacterial population exhibited complete red fluorescence conversion. These findings imply that AMP-17’s antibacterial mechanism likely involves coordinated modulation of both outer and inner membrane permeability.

### 3.8 PMF and ROS changes

**Figure 5:**
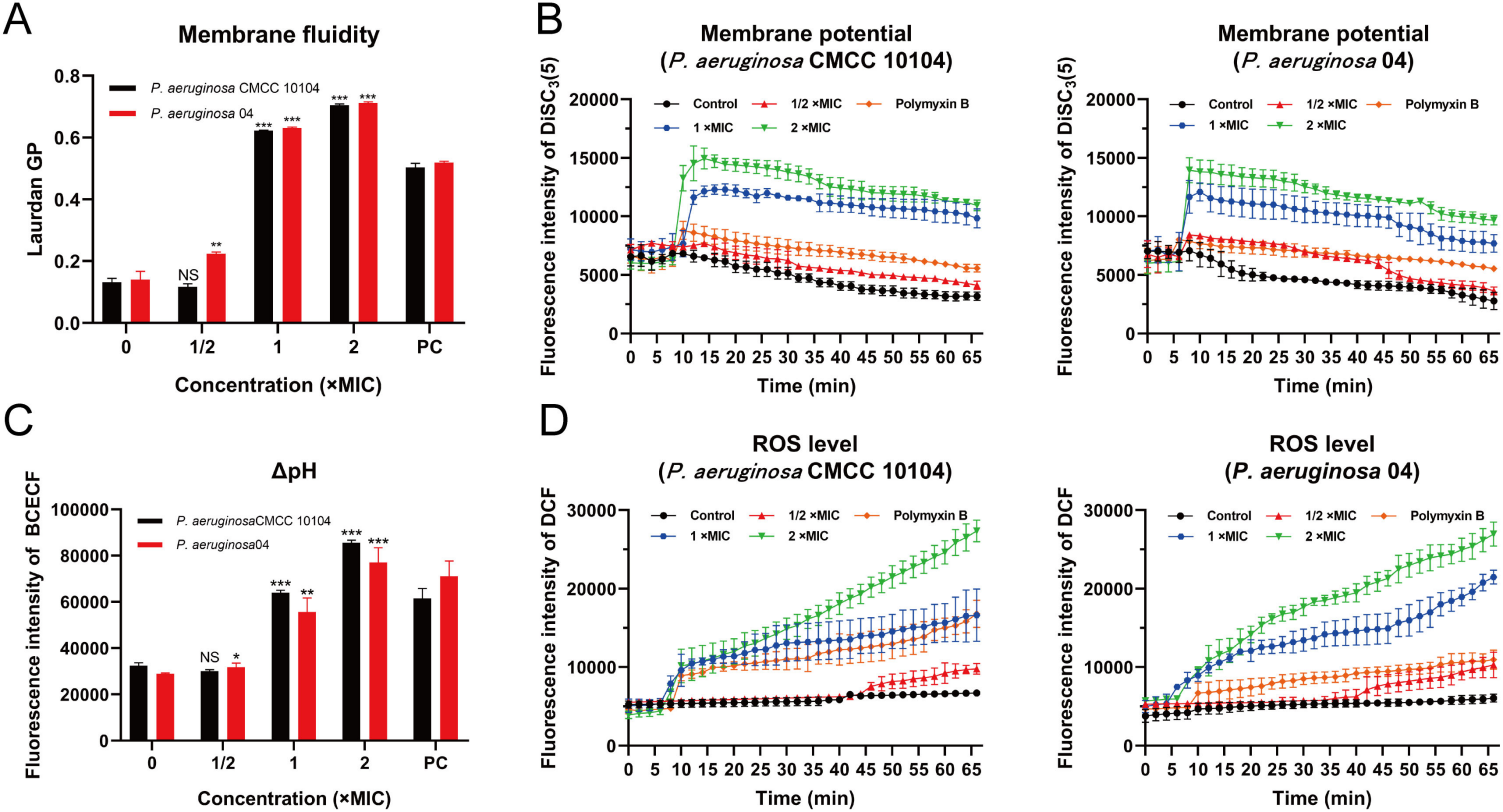
Effect of AMP-17 on PMF of *P. aeruginosa* CMCC 10104 and 04. **(A)** Effect of AMP-17 on membrane fluidity. **(B)** Effect of AMP-17 on the membrane potential of *P. aeruginosa* CMCC 10104 and 04. **(C)** Effect of AMP-17 on the ΔpH of *P. aeruginosa* CMCC 10104 and 04. **(D)** AMP-17 induced the accumulation of ROS in *P. aeruginosa* CMCC 10104 and 04. Compared with the control group, NS means non-significance, *P< 0.05, **P< 0.01, ***P< 0.001. PC, the positive control group.

Laurdan GP analysis revealed concentration-dependent elevation of generalized polarization values in AMP-17-treated groups (1 ×MIC and 2 ×MIC) relative to untreated controls, implying potential compound-mediated modulations in membrane fluidity (**Figure 5A**). This phenomenon appears consistent with previously documented alterations in membrane permeability.

Given the well-established correlation between membrane permeability changes and PMF dynamics, a critical bioenergetic parameter that integrates membrane potential (Δψ) and transmembrane proton gradient (ΔpH) to support bacterial survival, we systematically investigated these components. Membrane potential perturbations were evaluated using a DiSC_3_(5) fluorescent probe. Following co-incubation with *P. aeruginosa*, fluorescence baselines remained stable throughout the 5-minute equilibration period. Subsequent administration of AMP-17 at varying concentrations induced dose-responsive fluorescence intensity increases (**Figure 5B**), indicative of possible membrane depolarization events. For transmembrane proton gradient assessment, the BCECF-AM probe was implemented. This non-fluorescent compound undergoes intracellular esterase-mediated conversion to BCECF, a pH-sensitive fluorophore whose emission intensity demonstrated a positive correlation with AMP-17 concentrations (**Figure 5C**), suggesting potential disruption of proton gradient homeostasis.

In this study, intracellular ROS accumulation in *P. aeruginosa* cells was quantitatively assessed using the non-fluorescent probe DCFH-DA. Upon oxidation by ROS, DCFH-DA is converted to the highly fluorescent compound DCF, the fluorescence intensity of which was measured using a fluorescence microplate reader to determine ROS levels. Treatment with 2 ×MIC of AMP-17 resulted in a higher concentration of ROS compared to the 1 ×MIC group, with ROS levels increasing over time (**Figure 5D**). These findings suggest that AMP-17 induces ROS accumulation in *P. aeruginosa* cells and induces bacterial death.

### 3.9 AMP-17 promotes wound healing in a mice model

**Figure 6:**
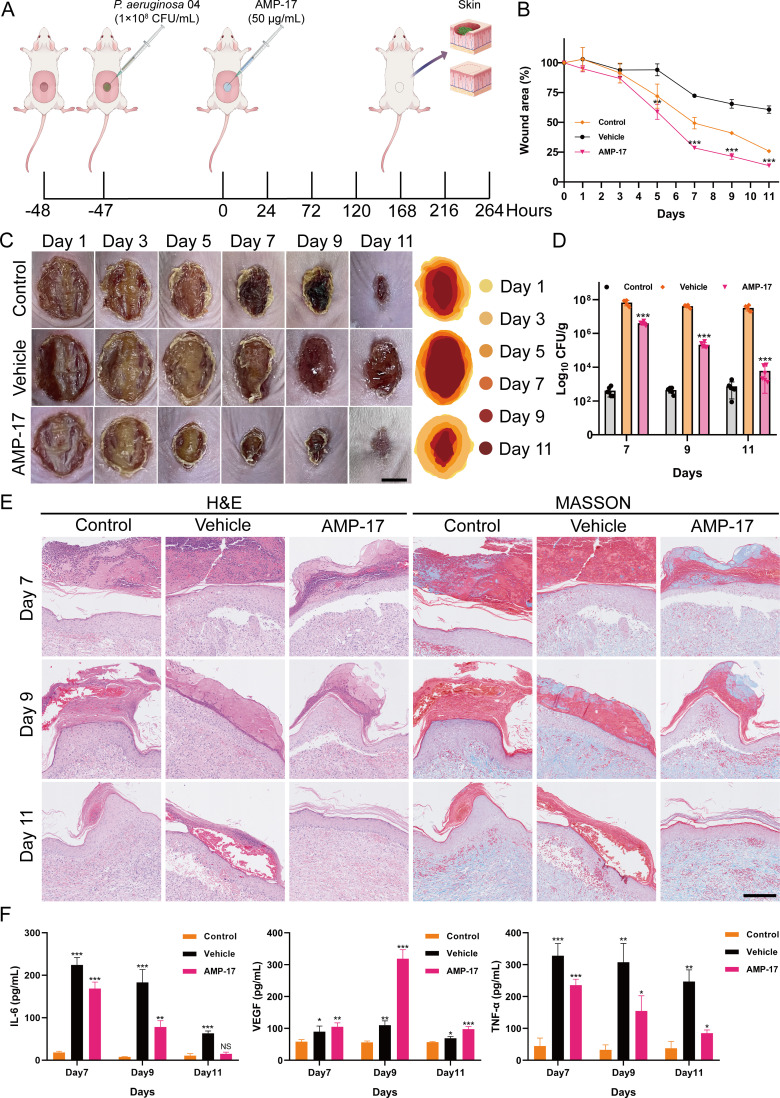
Effect of AMP-17 on wound healing *in vivo*. **(A)** Schematic illustration of the experimental design for the murine wound healing model. **(B)** Percent change in wound area over time. Compared with the vehicle group, **P< 0.01, ***P< 0.001. **(C)** Representative photographic images of wounds on days 1, 3, 5, 7, 9, and 11 post-injury. Scal, 0.5 cm. **(D)** Bacterial load of *P. aeruginosa* 04 in wound tissues, expressed as colony-forming units per gram (CFU/g). Compared with the vehicle group, ***P< 0.001. **(E)** Histopathological analysis of murine tissue sections stained with hematoxylin and eosin (H&E) and Masson’s trichrome. Scal, 200 μm. **(F)** Expression levels of inflammation-related cytokines in wound tissues. Compared with the control group, NS means non-significance, *P< 0.05, **P< 0.01, ***P< 0.001.

To evaluate the *in vivo* efficacy of AMP-17, a murine wound infection model was established. One hour after creating full-thickness skin wounds on the dorsum of BALB/c mice, the wounds were topically inoculated with 1×10^8^ CFU/mL of *P. aeruginosa* 04 (**Figure 6A**). At 48 hours post-wounding, the AMP-17 treatment group received a topical application of 50 μg/mL AMP-17, while the vehicle group was treated with an equal volume of PBS as a control. Wound tissues were harvested on days 7, 9, and 11 for analysis. In the absence of *P. aeruginosa* infection, the wound healing rate in the control group reached 74.18% by day 11, whereas the vehicle and AMP-17 group exhibited healing rates of 39.25% and 86.35%, respectively (**Figures 6B, C**). Bacterial load analysis revealed that AMP-17 treatment significantly reduced the wound bacterial burden by 1.22 Log_10_ CUF/g, 2.29 Log_10_ CUF/g, and 3.73 Log_10_ CUF/g on days 7, 9, and 11, respectively, compared to the model group (**Figure 6D**). Histopathological examination demonstrated that the AMP-17 group exhibited markedly reduced inflammatory cell infiltration and fibroblast proliferation, along with enhanced collagen deposition, compared to both the control and model groups (**Figure 6E**), and the expression of VEGF was increased, suggesting that AMP-17 promotes wound healing (**Figure 6F**). Furthermore, while the expression levels of pro-inflammatory cytokines IL-6 and TNF-α were significantly upregulated in the model group, AMP-17 treatment markedly downregulated their expression. Collectively, these results indicate that AMP-17 exhibits potent antimicrobial activity against P. aeruginosa 04 infection *in vivo* and promotes wound healing by suppressing infection-induced inflammatory responses.

## 4 Discussion

The escalating prevalence of multidrug-resistant *P. aeruginosa* strains, exacerbated by the misuse of antibiotics, has significantly complicated clinical treatment strategies (Aggarwal et al., 2024). Concurrently, the biofilm formation by *P. aeruginosa* impedes the efficacy of external drugs and immune responses, leading to persistent infection (Karruli et al., 2023). Traditional antibiotics exhibit diminished effectiveness against these resistant strains, underscoring the urgent need for novel antimicrobial agents. Antimicrobial peptides, as essential components of novel antibacterial agents, have emerged as promising therapeutic candidates due to their broad-spectrum antimicrobial activities against bacteria, fungi, and viruses, coupled with their unique mechanisms of action and low tendency for resistance development (Xuan et al., 2023).

In this study, we evaluated the *in vitro* antibacterial activity of AMP-17 against multi-drug resistant *P. aeruginosa*, determining MIC and MBC values of 25 μg/mL and 50 μg/mL, respectively. Growth curve analysis and bactericidal kinetics revealed that AMP-17 exhibits significant antibacterial and bactericidal effects, with activity positively correlated to the duration of exposure. In addition to their potent antimicrobial properties, the physiological stability of AMPs constitutes a key factor in their potential clinical development (Mwangi et al., 2023). Our experimental data indicate that AMP-17 maintains consistent anti-pseudomonal activity against *P. aeruginosa* when exposed to physiological salt concentrations and serum components, suggesting favorable characteristics for potential therapeutic applications.

Biofilm formation, a complex developmental process encompassing bacterial adhesion, aggregation, maturation, and subsequent dispersion (Guzman-Soto et al., 2021), represents a significant therapeutic challenge. Notably, *P. aeruginosa* biofilms demonstrate substantially enhanced antibiotic resistance compared to their planktonic counterparts (Cendra and Torrents, 2021). While current antimicrobial strategies predominantly target planktonic bacteria in acute infections, effective therapeutic options against established biofilms remain limited (Yu and Chua, 2020). Given the established correlation between bacterial motility and biofilm-associated pathogenicity (Kuhn et al., 2021), our findings that AMP-17 significantly inhibits *P. aeruginosa* motility suggest its potential to disrupt initial biofilm adhesion. Remarkably, AMP-17 demonstrated concentration-dependent antibiofilm activity, inhibiting biofilm formation at 1/2 × MIC and disrupting mature biofilms at MIC levels. These observations indicate that AMP-17 may interfere with critical biofilm developmental processes through inhibition of bacterial adhesion and aggregation. Although AMP-17 has demonstrated preliminary anti-biofilm activity against *P. aeruginosa in vitro*, further validation through dynamic or *in vivo* experimental systems is warranted to account for the complexity of physiological environments in future. Furthermore, considering the well-established role of pyocyanin as a key virulence factor in *P. aeruginosa* pathogenesis (Goncalves and Vasconcelos, 2021) - particularly in mediating host inflammatory responses and facilitating bacterial colonization (Thees et al., 2021) - our observation of AMP-17-mediated pyocyanin suppression provides mechanistic insights. This reduction in virulence factor production may contribute to the observed antibiofilm effects, suggesting a potential dual mechanism of action targeting both biofilm formation and virulence expression.

The interaction between AMPs and bacterial membranes constitutes a fundamental mechanism of their antimicrobial activity. The integration of membrane-disrupting compounds into lipid bilayers typically induces substantial alterations in membrane fluidity, culminating in the displacement of membrane proteins, efflux of intracellular components, and ultimately, bacterial cell death (Li et al., 2022; Saeed et al., 2022). Recent investigations have demonstrated that amphiphilic AMPs can enhance bacterial membrane permeability, triggering membrane depolarization and subsequent ROS generation (Shi et al., 2021). Our findings suggest that AMP-17 may exert its bactericidal effects through modulation of membrane fluidity properties. This membrane permeabilization is concomitant with the disruption of transmembrane potential and interference with PMF maintenance. These coordinated bioenergetic disturbances likely impair essential PMF-dependent cellular processes, ultimately leading to cell death. Furthermore, the antimicrobial efficacy of AMP-17 appears to be augmented through ROS-mediated mechanisms. Exposure to AMP-17 induces antimicrobial stress, stimulating intracellular ROS accumulation, which subsequently inflicts additional cellular damage, thereby compromising bacterial viability (Chen et al., 2023).

The skin barrier serves as the primary physical defense against pathogenic infections, with intact skin structure being crucial for maintaining organismal health. When the skin barrier function is compromised, pathogenic bacteria readily colonize the wound site and initiate infections (Uberoi et al., 2024). With the escalating prevalence of drug-resistant bacterial infections, the limitations of conventional antibiotic therapies have become increasingly apparent. In addressing wound infections caused by drug-resistant bacteria, antimicrobial peptides have emerged as a focal point in current research as next-generation antimicrobial agents (Haidari et al., 2023). Previous studies have demonstrated that AMPs significantly inhibit bacterial colonization in skin wounds and promote tissue repair processes (Satapathy et al., 2024). Furthermore, AMPs have been shown to possess multiple beneficial effects, including promoting wound healing, inhibiting bacterial colonization, and reducing inflammatory responses (Miao et al., 2021). During the complex physiological process of wound healing, VEGF plays a pivotal regulatory role, while the dynamic changes in pro-inflammatory cytokines IL-6 and TNF-α significantly influence the healing progression (Hirano, 2021; Raziyeva et al., 2021; de Souza et al., 2022). Our study demonstrates that AMP-17 not only exhibits remarkable therapeutic effects on wound healing by effectively inhibiting the colonization of drug-resistant bacterial, but also potentially promotes tissue repair through a dual regulatory mechanism: upregulating VEGF expression and collagen deposition while downregulating IL-6 and TNF-α levels to mitigate inflammatory responses, thereby synergistically enhancing the wound healing process (**Figure 6F**).

**Figure 7:**
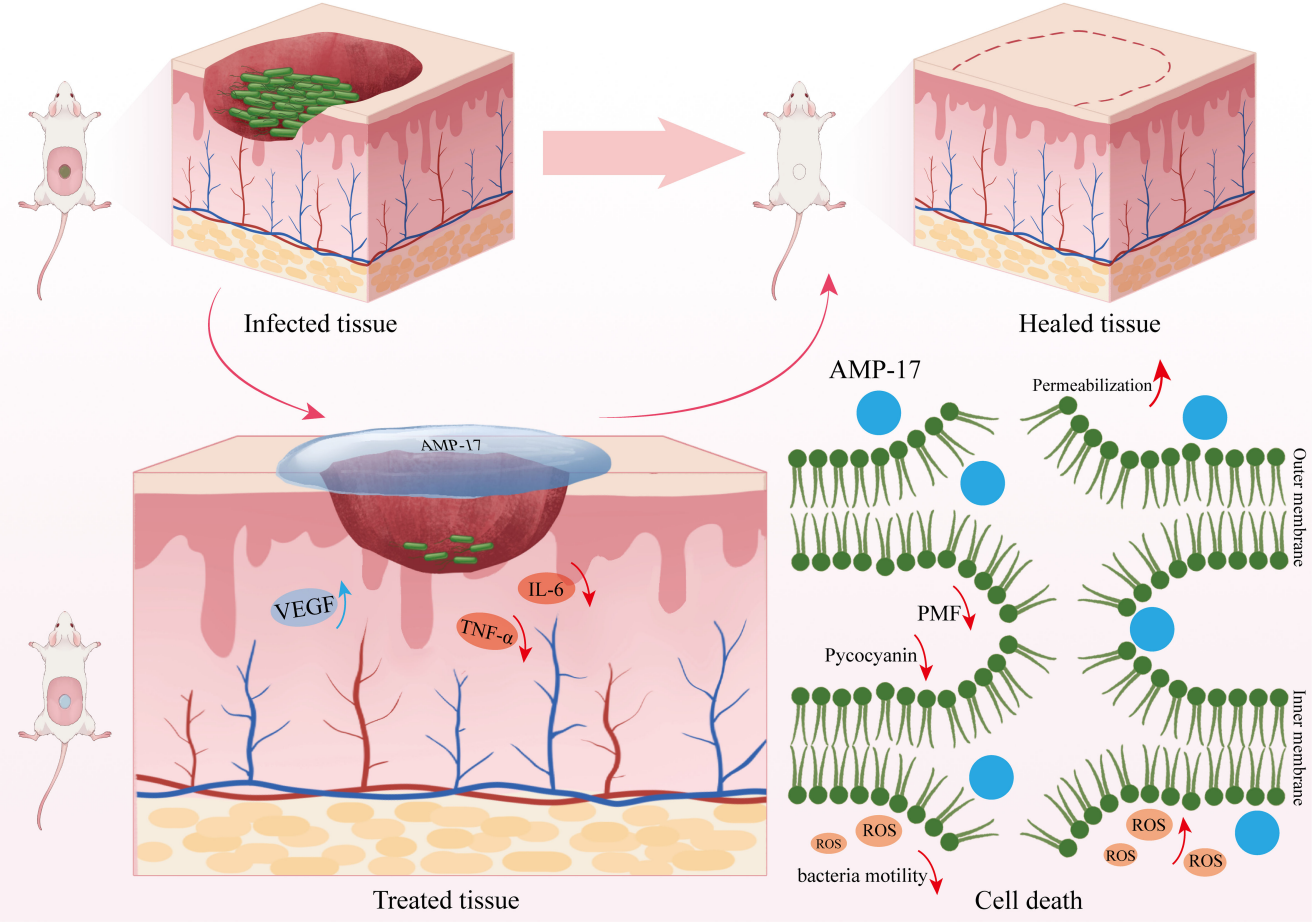
Mechanism of AMP-17 against *P. aeruginosa* and its promotion of wound healing. AMP-17 exerts its antimicrobial activity against *P. aeruginosa* through multiple mechanisms, including increased membrane permeability and fluidity, reduced pyocyanin production, accumulation of intracellular ROS, and disruption of PMF. Additionally, AMP-17 promotes wound healing by enhancing VEGF and collagen expression and downregulating pro-inflammatory cytokines IL-6 and TNF-α in skin tissues.

In summary, our study reveals that AMP-17 exerts its antimicrobial and wound-healing promoting effects through multiple mechanisms. Regarding its antimicrobial action, AMP-17 induces bacterial cell death by disrupting membrane fluidity and increasing the permeability of both inner and outer membranes in *P. aeruginosa*. This process leads to cytoplasmic leakage, dissipation of proton motive force, and subsequent deformation and rupture of the cellular membrane. Furthermore, AMP-17 promotes the generation and accumulation of ROS, exacerbating cellular damage and ultimately resulting in bacterial death. As for its wound-healing properties, AMP-17 enhances the expression of VEGF and deposition of collagen while downregulating the levels of pro-inflammatory cytokines IL-6 and TNF-α in wound tissues, thereby promoting the wound healing process (**Figure 7**).

The escalating prevalence of antibiotic-resistant bacterial strains and resistance genes has rendered the identification of viable alternatives to conventional antibiotics an urgent priority in contemporary medicine. AMPs have emerged as promising candidates due to their superior safety profile, minimal residual effects, and low propensity for resistance development. Their distinctive antimicrobial mechanisms exhibit considerable potential in combating antibiotic resistance. Notably, AMP-17 demonstrates salt tolerance, serum stability, and membrane-disruptive activity, endowing it with advantages over traditional antibiotics. Future investigations should focus on validating AMP-17’s pharmacokinetic properties, long-term toxicological safety, and therapeutic efficacy across diverse infection models. Furthermore, elucidating its synergistic potential with other antimicrobial agents will be paramount for clinical translation. These collective efforts may ultimately establish AMP-17 as a cornerstone in our antimicrobial armamentarium against drug-resistant pathogens.

## Data Availability

The raw data supporting the conclusions of this article will be made available by the authors, without undue reservation.
